# Long noncoding RNAs involve in resistance to *Verticillium dahliae*, a fungal disease in cotton

**DOI:** 10.1111/pbi.12861

**Published:** 2017-12-21

**Authors:** Lin Zhang, Maojun Wang, Nannan Li, Honglei Wang, Ping Qiu, Liuling Pei, Zheng Xu, Tianyi Wang, Erlin Gao, Junxia Liu, Shiming Liu, Qin Hu, Yuhuan Miao, Keith Lindsey, Lili Tu, Longfu Zhu, Xianlong Zhang

**Affiliations:** ^1^ National Key Laboratory of Crop Genetic Improvement Huazhong Agricultural University Wuhan Hubei China; ^2^ Integrative Cell Biology Laboratory School of Biological and Biomedical Sciences Durham University Durham UK

**Keywords:** cotton, lncRNA, *Verticillium dahliae*, genomewide expression profile, virus‐induced gene silencing, broad resistance

## Abstract

Long noncoding RNAs (lncRNAs) have several known functions in plant development, but their possible roles in responding to plant disease remain largely unresolved. In this study, we described a comprehensive disease‐responding lncRNA profiles in defence against a cotton fungal disease *Verticillium dahliae*. We further revealed the conserved and specific characters of disease‐responding process between two cotton species. Conservatively for two cotton species, we found the expression dominance of induced lncRNAs in the Dt subgenome, indicating a biased induction pattern in the co‐existing subgenomes of allotetraploid cotton. Comparative analysis of lncRNA expression and their proposed functions in resistant *Gossypium barbadense* cv. ‘7124’ versus susceptible *Gossypium hirsutum* cv. ‘YZ1’ revealed their distinct disease response mechanisms. Species‐specific (LS) lncRNAs containing more SNPs displayed a fiercer inducing level postinfection than the species‐conserved (core) lncRNAs. Gene Ontology enrichment of LS lncRNAs and core lncRNAs indicates distinct roles in the process of biotic stimulus. Further functional analysis showed that two core lncRNAs, *GhlncNAT‐ANX2‐* and *GhlncNAT‐RLP7‐*silenced seedlings, displayed an enhanced resistance towards *V. dahliae* and *Botrytis cinerea*, possibly associated with the increased expression of *LOX1* and *LOX2*. This study represents the first characterization of lncRNAs involved in resistance to fungal disease and provides new clues to elucidate cotton disease response mechanism.

## Introduction

The transcriptional landscape in eukaryotes has been extensively studied using RNA sequencing (RNA‐seq) and reveals that RNA molecules are transcribed ranging from protein‐coding mRNAs to noncoding transcripts (Berretta and Morillon, 2009; Chekanova *et al*., 2007; Kapranov *et al*., 2007; Ponting *et al*., 2009; Sanchez‐Leon *et al*., 2012; Yamada *et al*., 2003; Zhu *et al*., 2014). Noncoding RNAs are classified into two types, containing either short sequences (<200 nt) or long noncoding RNAs (lncRNAs, longer than 200 nt) (Bertone *et al*., 2004; Cabili *et al*., 2011; Guttman *et al*., 2009; Wang *et al*., 2014a; Zhou *et al*., 2014). lncRNAs can in turn be classified into long intergenic noncoding RNAs (lincRNAs), natural antisense transcripts (NATs) and intronic RNAs (incRNAs) based on genome location (Chen, 2012; Dogini *et al*., 2014; Ma *et al*., 2014; Ponting *et al*., 2009; Rinn and Chang, 2012; Wang *et al*., 2015a; Weick and Miska, 2014). Studies of the biological roles of lncRNAs are challenging because of their diverse expression and regulation patterns across a wide range of cells and tissues (Orom and Shiekhattar, 2011). lncRNAs realized their functions mostly as signals, decoys, guides and scaffolds (Wang and Chang, 2011).

Although a large number of lncRNAs have been identified from sequencing data, only a few lncRNAs are functionally well characterized in plants. Two lncRNAs from *Arabidopsis*,* COOLAIR* and *COLDAIR*, have been characterized from *FLOWERING LOCUS C (FLC)* that acts as a floral repressor (Heo and Sung, 2011; Swiezewski *et al*., 2009). In rice, *LONG‐DAY‐SPECIFIC MALE‐FERTILITY‐ASSOCIATED RNA* (*LDMAR*), exerting like a structure lncRNA, regulates photoperiod‐sensitive male sterility (PSMS) (Ding *et al*., 2012). In *Medicago truncatula*, the lncRNA *Enod40* involves symbiotic interactions with soil rhizobia in nodule formation by regulating the relocalization of a nuclear RBP (Campalans *et al*., 2004). Several lncRNAs responding to *Fusarium oxysporum* infection have been identified in *Arabidopsis*, but with unknown function (Zhu *et al*., 2014). Recently, lncRNA ELF18‐INDUCED LONG‐NONCODING RNA1 (ELENA1) identified in *Arabidopsis* enhanced the resistance against *Pseudomonas syringe* via interacting with Mediator subunit 19a to regulate *PR1* (Seo *et al*., 2017). These findings highlight the essential function and increasing attention of lncRNAs in plant biology and in controlling important agronomic traits.

Cotton (*Gossypium* spp.) has long been widely cultivated for its renewable textile fibre and seeds oil. More than 90% of cultivated cotton was allotetraploid, which originated from the accidently merging of two progenitor donors with A genome and D genome, respectively (much like modern *G. arboretum* and *G. raimondii*), 1–2 million years ago (Wendel and Cronn, 2003; Wendel *et al*., 2012; Zhang *et al*., 2015b). It takes thousands of years for human to domesticate cotton from wild to modern cultivated cotton, which produces the spinnable, fine white fibres (Wang *et al*., 2017). However, China now faces the huge economic loss resulting from the sharply decreased cotton yield and quality, which were destroyed by *Verticillium* wilt (VW). VW is caused by soil‐borne fungus *Verticillium dahliae*, which worldwide invades more than 400 plant species hosts (Li *et al*., 2017; Zhang *et al*., 2016). This disease will lead to chlorosis and wilt of leaves or defoliation, the browning of vascular and even death ultimately (Li *et al*., 2014; Xu *et al*., 2011). It has been the major challenge for cotton and deserves enormous researches to control efficiently.

Plants possess a multilayered immune system to counteract pathogens through both constitutive and inducible defences, such as physical and chemical barriers, pattern recognition receptors (PRRs) and resistance genes (R genes) encoding proteins containing a nucleotide‐binding site (NBS) with leucine‐rich repeats (LRRs) (Bent and Mackey, 2007; Jones and Dangl, 2006; Yang *et al*., 2013). However, the recognition of apoplastic pathogen effectors is mediated by receptor‐like proteins (RLPs), such as Ve1 (de Jonge *et al*., 2012). In addition, some plant hormones, such as jasmonic acid (JA), salicylic acid (SA) and ethylene (ET), act as immunity signal molecules (Bari and Jones, 2009). SA can activate effective defence responses against hemibiotrophs and biotrophs, which are important for the establishment of systemic acquired resistance (SAR) (Dempsey and Klessig, 2012; Yang *et al*., 2015). JA functions with ethylene to activate resistance against necrotrophic pathogens (Cacas *et al*., 2016; Thaler *et al*., 2004).

Contrasted with protein‐coding genes, immunity‐related lncRNAs are less well documented in plant immunity. However, advanced sequencing data will unveil profiles of lncRNAs and provide new insights and promising lncRNA candidates in this area. There were some small RNAs identified related to cotton defence againt *V. dahliae* (He *et al*., 2014; Yin *et al*., 2012), but information related to lncRNAs in cotton was restricted to fibre development (Wang *et al*., 2015b). The availability of the complete genome sequences of *Gossypium barbadense* and *Gossypium hirsutum* has made it possible to conduct a genomewide comparative analysis of lncRNAs associated with disease response (Yuan *et al*., 2015; Zhang *et al*., 2015b).

Here, we reported the first charaterization of resistance‐associated lncRNAs in two distinct cotton species, *G. barbadense* (which is resistant to VW) and *G. hirsutum* (which is susceptible). We showed that the different resistance responses were caused by the genomic divergence between the two tetraploid cotton species. We related disease response to lncRNA profile and identified functional lncRNAs in the cotton immune response following infection by *V. dahliae*.

## Results

### Identification and characterization of lncRNAs in cotton root

**Figure 1:**
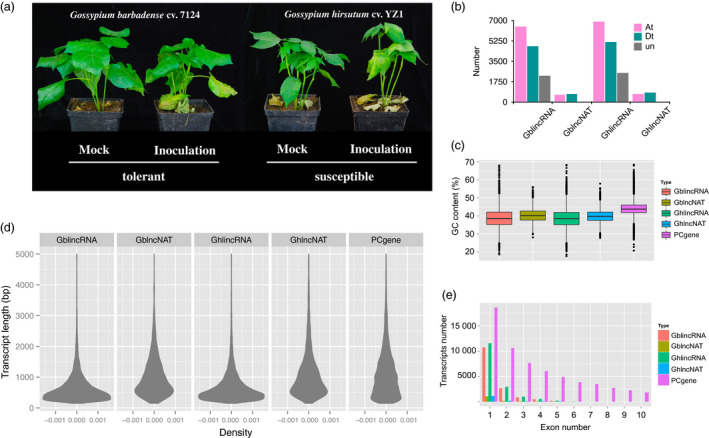
Identification and characterization of long noncoding RNAs (lncRNAs) in *Gossypium barbadense* and *Gossypium hirsutum*. (a) Characterization of resistance to *Verticillium dahliae* in *G*. *barbadense* and *G. hirsutum*. (b) Distribution of long intergenic noncoding RNAs (lincRNAs) and long noncoding natural antisense transcripts (lncNATs) in the At subgenome, Dt subgenome and ungrouped scaffolds separately for *G. barbadense* (Gb) and *G.  hirsutum* (Gh). (c) The GC content of different genes in cotton. (d) Density plot showing transcript length distribution of lincRNAs, lncNATs and protein‐coding genes. (e) Exon number distribution of lincRNAs, lncNATs and protein‐coding genes.

*Verticillium dahliae* primarily infects cotton from roots, and thus, we are interested in analysing lncRNAs profiles in roots. Two cotton species, *G*. *barbadense* (resistant) and *G. hirsutum* (susceptible), were inoculated for sequencing root samples (Figures 1a and S1). We generated 12 high‐depth transcriptomes consisting of more than 1.5 billion clean reads, of which six were produced from *G*. *barbadense* and the other six were produced from *G. hirsutum* (Figure S1). We used an integrated approach (see Experimental procedures) to identify high‐confidence lncRNAs for each cotton species. Four classes of lncRNAs were identified, and the majority of them were long intergenic noncoding RNAs (lincRNAs) and long noncoding natural antisense transcripts (lncNATs). In total, there were 13 452 loci of lincRNAs and 1297 loci of lncNATs in *G*. *barbadense*, and 14 547 loci of lincRNAs and 1406 loci of lncNATs in *G. hirsutum* (Table 1). The numbers of lincRNAs in the At subgenome were larger than those in the Dt subgenome, for *G*. *barbadense* and *G. hirsutum* (Figure 1b). However, the numbers of lncNATs in the At and Dt subgenome were similar (Figure 1b).

**Table 1:** Number of major types of lncRNAs

Cotton species	lincRNA	lncNAT	Sense	Intronic
*Gossypium barbadense*	13 452	1297	260	200
*Gossypium hirsutum*	14 547	1406	262	198

lincRNA, long intergenic noncoding RNAs; lncNAT, long noncoding natural antisense transcripts.

To prove that the full transcriptomes from libraries with only removal of rRNAs contain more complete noncoding message, we also sequenced two stranded libraries in which only mRNAs with poly(A) tails were retained for comparison. As expected, we found more lncRNAs were identified in libraries with the removal of rRNAs than in the regular stranded libraries following the same identification procedure (Table S1). For example, more than 32% of lincRNAs and 159% (namely 1.6‐fold) of lncNATs were identified in the full transcriptome of sample Y12m (Table S1).

GC content, which reflects the biased intergenomic nonreciprocal DNA exchanges (Guo *et al*., 2014), was investigated for lncRNAs. The result showed that lincRNAs and lncNATs exhibited lower GC content than protein‐coding genes in both cultivars (Figure 1c). lincRNAs were found to have a lower GC content than lncNATs. There was no difference in GC content between *G*. *barbadense* and *G. hirsutum* both for lincRNAs and lncNATs.

The average length of protein‐coding transcripts (1180 bp) was similar to the sequence length of lncNATs (1061 bp in *G. barbadense*, 1150 bp in *G. hirsutum*), but was larger than those of lincRNAs both in *G. barbadense* and *G. hirsutum* (678 bp in *G. barbadense*, 729 bp in *G. hirsutum*). lncNATs and protein‐coding transcripts exhibited a similar trend of length distribution. In contrast, lincRNAs showed an earlier peak primarily because of the large population of short sequences (Figure 1d). Analysis of exon number distribution revealed that all types of single‐exonic transcripts represented the largest proportion (Figure 1e). The ratio of single‐exonic lncRNAs was extremely high, especially for lncNATs in *G. barbadense* (72.6%). However, single‐exonic protein‐coding transcripts had the lowest ratio (29.9%).

### Biased expression of lncRNAs upon infection in co‐existing subgenomes

**Figure 2:**
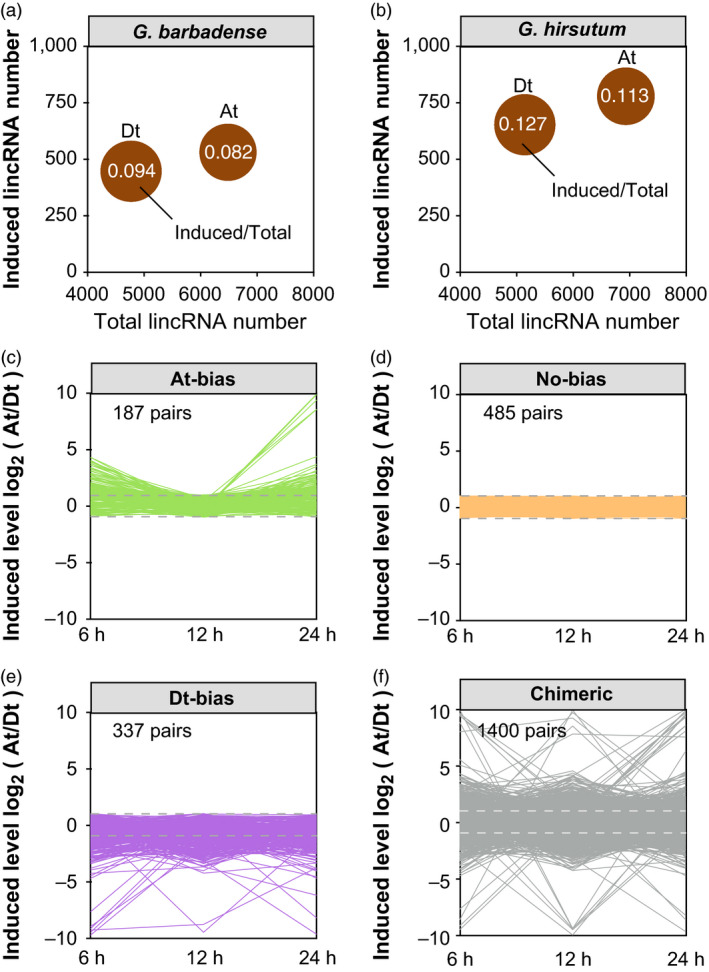
Comparison of pathogen response for lncRNAs in subgenomes. (a) Ratio of differentially induced lncRNAs in *Gossypium barbadense*. *X*‐axis represents the total number and *Y*‐axis represents the differentially induced number of lncRNAs. (b) Ratio of differentially induced lncRNAs in *Gossypium hirsutum*. (c) The category of At‐bias induced lncRNAs from *G. barbadense*. Grey dashed lines mean the cut**‐**off of bias induced expression (|log2(At/Dt)| = 1). (d) The category of No‐bias induced lncRNAs. (e) The category of Dt‐bias induced lncRNAs. (f) The category of Chimeric induced lncRNAs.

Homoeologous expression bias was found to exist widely in allopolyploids species, presenting underexplored scale in transcriptomic diversity and evolution process (Yoo *et al*., 2013; Yuan *et al*., 2015). We found that, in *G. barbadense*, the induced ratio of lincRNAs from the Dt subgenome is 0.094, while the ratio from the At subgenome was 0.082 (Figure 2a). In *G. hirsutum*, induced ratios of lincRNAs from the Dt subgenome and the At subgenome were 0.127 and 0.113, respectively (Figure 2b).

Using the reciprocal best match alignment, there are 1757 homoeologous lincRNA pairs between the At subgenome and Dt subgenome in *G. barbadense*. We obtained 187 lincRNAs pairs for At‐biased induced expression and 337 lincRNAs pairs for Dt‐biased induced expression (Figure 2c,e). We also found that 485 pairs showed no‐biased expression and the other 1400 pairs showed a chimeric expression pattern (Figure 2d,f). Simultaneously in *G. hirsutum*, among 2026 homoeologous lincRNA pairs, we found 223 lincRNAs pairs of At‐biased, 352 pairs of the Dt‐biased induced pattern after inoculation.

To further examine this biased distribution of disease response loci, we collected evidence as complete as possible from prior quantitative trait locus (QTL) mapping results for *Verticillium wilt* resistance and found the biased distribution in two subgenomes (Total number At: 76; Dt: 97; summarized in Figure S2 and detailed in Table S2) (Fang *et al*., 2013a,b, 2017b; Jiang *et al*., 2009; Li *et al*., 2017; Wang *et al*., 2008, 2014b; Yang *et al*., 2008; Zhang *et al*., 2014a, 2015a; Zhao *et al*., 2014; Zhiyuan *et al*., 2013).

Previous studies had shown that neighbour protein‐coding genes might have functional connections with lncRNAs and might have similar expression profiles (Engreitz *et al*., 2016; Luo *et al*., 2016; Wang *et al*., 2015b; Wierzbicki *et al*., 2008). The possibility was measured by calculating the Pearson correlation coefficients (*r*_p_) for three groups: lincRNAs and their adjacent protein‐coding genes (lincRNA‐PCgene: 5928 pairs); lncNATs and their paired protein‐coding genes on opposite strand (lncNAT‐PCgene: 1407 pairs); protein‐coding genes and nearest protein‐coding genes (PCgene‐PCgene pairs: randomly selected 5000 pairs). In contrast with the random PCgene pairs, there were higher positive correlations in identified lncRNA‐associated pairs (Figure S3). For instance, we noticed the high ratio of positively correlated lincRNA–PCgene pairs (10% vs 5%; *r*_p_ > 0.8) and lncNAT–PCgene pairs (12% vs 6%; *r*_p_ > 0.8).

Gene Ontology (GO) enrichment of lncRNAs was putatively conducted according to the functional annotations of neighbour protrein‐coding genes. The results displayed that At‐biased induced lincRNAs were enriched in kinase activator activity, fructose‐bisphosphate aldolase activity and structure‐specific DNA binding (Table 2). Nevertheless, Dt‐biased induced lincRNAs were enriched in signal transducer activity, MAP kinase activity and superoxide dismutase copper chaperone activity. This illustrates distinct disease response mechanisms of two divergent subgenomes, which may have resulted from asymmetric evolution during allopolyploid formation and long‐term domestication (Wang *et al*., 2017; Zhang *et al*., 2015b).

**Table 2:** The Gene Ontology of At‐ and Dt‐biased lncRNAs

Type	GO‐ID	Term	*P*‐value
At‐bias	GO:0019209	Kinase activator activity	2.33E‐03
	GO:0003690	Double‐stranded DNA binding	2.66E‐03
	GO:0030983	Mismatched DNA binding	2.66E‐03
	GO:0004332	Fructose‐bisphosphate aldolase activity	1.41E‐02
	GO:0043566	Structure‐specific DNA binding	1.75E‐02
Dt‐bias	GO:0004871	Signal transducer activity	6.84E‐03
	GO:0008173	RNA methyltransferase activity	7.45E‐03
	GO:0004707	MAP kinase activity	1.90E‐02
	GO:0005057	Receptor signalling protein activity	1.90E‐02
	GO:0016532	Superoxide dismutase copper chaperone activity	2.25E‐02

### Comparison of pathogen‐induced expression profiles of lncRNAs in two cotton species

**Figure 3:**
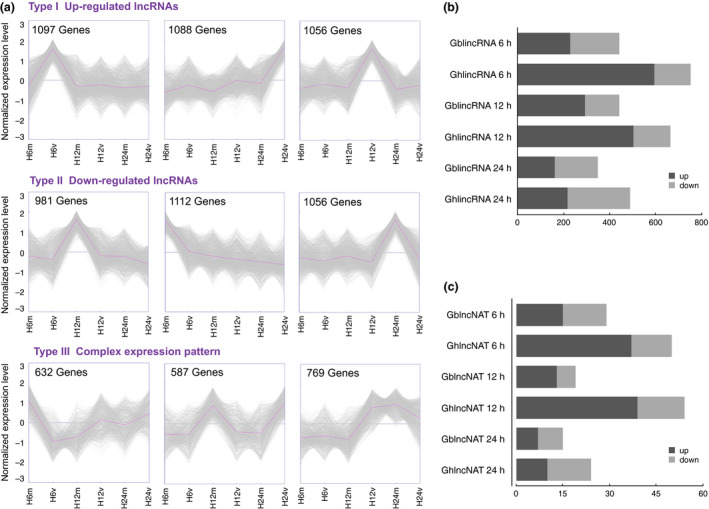
The global expression profiles of lncRNAs and distribution of differentially expressed lncRNAs. (a) Clusters of expressed lncRNAs in *Gossypium barbadense* developed by *K*‐means. ‘6’, ‘12’ and ‘24’ mean hours postinfection. ‘m’ and ‘v’ mean mock and seedling roots inoculated with *Verticillium dahliae* V991. (b) The distribution of differentially induced lincRNAs in two different cottons for each time point. (c) The distribution of differentially induced lncNATs.

The global expression patterns of lncRNAs in *G. barbadense* and *G. hirsutum* were, respectively, found to fall into three classes, as determined by a *K*‐means method (Figures 3a and S4). Type I and Type II clusters represent positively and negatively induced lncRNAs, respectively. Type III represents the complex expression patterns during pathogen infection. For instance, there were 632 lncRNAs, which were down‐regulated in 6 h postinfection and then slightly up‐regulated in later two time points (Figure 3a).

Intriguingly, we found distinct numbers of differentially expressed lncRNAs in two cotton species during the invasion of pathogens (*P*‐value <0.05; log2 ratio of 1). There were a total of 1236 and 1907 differentially expressed lincRNAs in *G. barbadense* and *G. hirsutum,* respectively (Figure 3b). The up‐regulated lincRNAs occupied a large proportion (*G. hirsutum*: 69%; *G. barbadense*: 56%). In addition, there were 63 and 128 differentially expressed lncNATs in *G. barbadense* and *G. hirsutum* separately (Figure 3c). In 12 h postinfection, the number of differentially expressed lncNATs in *G. hirsutum* was even twice of that in *G. barbadense* (Figure 3c). It seemed that more lncRNAs were differentially expressed in susceptible species, which suggests fiercer disease response.

**Figure 4:**
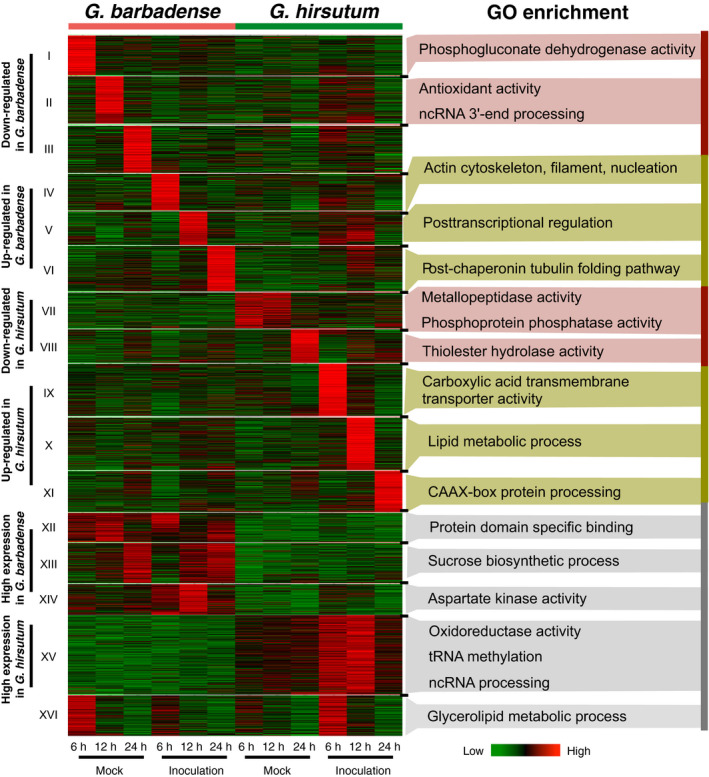
The comparison of induced pattern for lncRNAs in two different cotton cultivars. All expressed homologous lncRNA pairs between *Gossypium barbadense* and *Gossypium hirsutum* were clustered into 16 groups (I to XVI). Gene ontology (GO) terms are indicated by significant *P* values (*P *<* *0.01) for each cluster.

To compare the potential functions of lncRNAs between two different cotton species, the homologous lncRNAs between two cottons were identified by reciprocal BLAST alignment with the best hit. The differentially expressed homologous lncRNAs (3411 pairs) were divided into 16 groups (I to XVI) (Figure 4). Groups I to III and VII to VIII contained up‐regulated lncRNAs in *G. barbadense* and *G. hirsutum* separately; Groups IV to VI and IX to XI exhibited a down‐regulated pattern; Group XII to XIV showed a high level of expression in *G. barbadense* but a low level of expression in *G. hirsutum*; Group XV displayed a low level of expression in *G. barbadense* but a high level of expression in *G. hirsutum*; and Group XVI had a complex expression pattern, which was distinct from the other groups. GO enrichment analysis was conducted to infer the potential biology processes of lncRNAs for all groups except for Group III (*P*‐value < 0.01). For instance, Group II was enriched in antioxidant activity and ncRNA 3′‐end processing (Figure 4).

### Characterization of species‐conserved and species‐specific lncRNAs in *G*. *barbadense* and *G. hirsutum*

**Figure 5:**
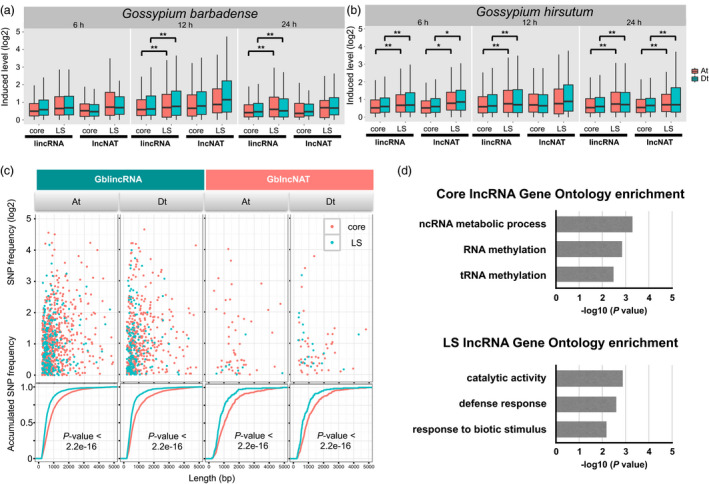
Characterization of core and specific lncRNAs. (a) The charts show changes in the induced expression levels (log2‐transformed FPKM) of different classes of core/lineage‐specific (LS) lncRNAs in *Gossypium barbadense* at three induced stages, 6 h postinfection (hpi), 12 hpi, 24 hpi. (b) Expression change of core/LS lncRNAs in *Gossypium hirsutum*. (c) SNP distribution of lineage‐specific (LS) lncRNAs and core lncRNAs. Scatter plot showing the correlation between SNP frequency and length of lncRNAs in *G. barbadense*. Significant levels of distribution divergence are indicated as *P* values. (d) Gene ontology enrichment analysis of neighbour protein‐coding genes of core lncRNAs and LS lncRNAs (*P *<* *0.01).

Genetic variation is required for rapid adaptation and evolution in the battle between plants and pathogens (de Jonge *et al*., 2013), which is expected to be reflected in lineage‐specific (LS) genomic regions. To explore whether the LS lncRNAs contribute to pathogen resistance, we compared LS lncRNAs with core lncRNAs, that is those common between cotton species. We identified 9443 unique loci of core lncRNAs in *G. barbadense* and 9937 unique loci in *G. hirsutum*. LS lncRNAs were also identified in *G. barbadense* (3943 unique loci) and *G. hirsutum* (5183 unique loci) (Table 3). Intriguingly, we found that a higher ratio of LS lncRNAs was differentially induced compared with core lncRNAs in both cultivars (Table 3). We also found that LS lincRNAs showed higher expression levels than core lincRNAs in both cultivars (Wilcoxon rank sum test, *, *P*‐value *< *0.01; **, *P*‐value *< *0.001; Figure 5a,b), except at 6 h postinfection (6 hpi). LS lncNATs in *G. hirsutum* also exhibited a significantly stronger pathogen induction, but no such significant difference was seen in *G*. *barbadense* (Figure 5a,b). These suggest that LS lncRNAs have greater expression changes towards pathogen infection than core lncRNAs.

**Table 3:** The identification of core and lineage‐specific (LS) lncRNAs

Classification	H core	H LS	Y core	Y LS
Total number	9443	3943	9937	5183
Induced number	565	514	975	725
Induced ratio	6%	12%	9%	12%

H, *Gossypium barbadense;* Y, *Gossypium hirsutum;* Core, Conserved sequence between two cotton species; LS, Lineage‐specific sequence between two cotton species.

To further elucidate evolution force of species‐conserved and species‐specific lncRNAs, the existence frequency of transposable elements (TEs) and polymorphic single‐nucleotide polymorphisms (SNP) was computed. TEs have long been recognized as a driving force for genome variation. To explore whether TEs contribute to the evolution of species‐specific lncRNAs, we calculated the occupation of TEs in LS lncRNAs and core lncRNAs. Unexpectedly, there was no obvious difference in TE distribution between LS and core lncRNAs in gene body and genic flanking regions (Figure S5). Therefore, the genetic variation was not mainly caused by TE insertions. In addition, SNP frequency in LS and core regions was calculated according to resequencing data using 58 *G. hirsutum* accessions and 70 *G. barbadense* accessions (Fang *et al*., 2017a; Wang *et al*., 2017). It was found that SNP frequency was consistently higher in LS lncRNAs than core lncRNAs in all comparisons (*P*‐value < 2.2e‐16) (Figures 5c and S6). These results indicated that SNP widely contributed to the variation of LS lncRNAs and might evolve more rapidly than core lncRNAs.

GO enrichment analysis (*P*‐value < 0.01) showed that core lncRNAs were enriched in ‘ncRNA metabolic process’ and ‘RNA methylation’ (Figure 5d). LS lncRNAs were preferentially enriched in ‘defence response process’ and ‘response to biotic stimulus’ (Figure 5d).

### Pairs of lncRNAs and neighbour genes and their expressions after inoculation

We collected differentially expressed gene pairs between protein‐coding genes and neighbour lncRNAs after inoculation for further functional identification. These pairs were divided into two groups, lincRNA/protein‐coding gene pairs and lncNAT/protein‐coding gene pairs. A total of 63 pairs of lincRNA/protein‐coding genes and 29 pairs of lncNAT/protein‐coding genes were identified (Figure S7a,b). We found that a large number of gene pairs were enriched in plant–pathogen interaction pathways, plant hormone signal transduction and starch and sucrose metabolism (Figure S7c,d), suggesting their functional implications in responding to *V. dahliae* infection. Genes participated in plant–pathogen interaction were selected for further validation.

Antisense expression is enriched when genes respond to environmental factors and stresses (Luo *et al*., 2016; Qi and Arkin, 2014; Xu *et al*., 2011). Then, the expression of lncNATs and neighbour paired protein‐coding genes was investigated. It was found that their expression patterns were complex following infection, including reverse, similar or nonrelated patterns in the two species (Figure S8). For instance, Gh_A03G1709 and its paired lncNAT (XLOC_005731) showed a similar induced pattern in *G. hirsutum*, with both of them being up‐regulated during pathogen invasion (Figure S8).

To validate the expression patterns of ten pairs from previously identified 29 pairs of lncNATs and the associated protein‐coding genes (Figure S9), qRT‐PCR experiment of protein‐coding gene Gh_D06G1866 (named P2) and its overlapping lncNAT XLOC_051276 (named L2) were performed in both cotton cultivars (Figure S9). Expression of more genes was identified by qRT‐PCR, including Gh_A01G1977 and its paired lncNAT XLOC_002524 (named P3 and L3), Gh_A03G0544 and XLOC_006187 (named P4, L4), Gh_A08G0154 and XLOC_019529 (named P6, L6), Gh_D08G1915 and XLOC_056034 (named P9, L9), Gh_D03G0546 and XLOC_040782 (named P10, L10), Gh_A03G1307 and XLOC_006730 (named P11, L11), Gh_A04G1172 and XLOC_007816 (named P12, L12), Gh_D05G3796 and XLOC_081611 (named P14, L14) and Gh_A13G0172 with XLOC_033015 (named P15, L15) (Figure S9). The majority (96%) of qRT‐PCR results showed a strong correlation (*r* = 0.8) with the transcriptome sequencing data (Figure S9).

### Functional candidate lncRNAs in resistance to *V. dahliae*

To annotate candidate genes that were associated with disease‐induced response, we adopted a phylogenetic approach using known homologous genes in *Arabidopsis*. CrRLK1L family RLKs are regulated by the steroid hormones brassinosteroids including several important receptor‐like kinase genes, such as *ANXUR2* (*ANX2*), *ANXUR1* (*ANX1*) and *FERONIA* (*FER*) (Lindner *et al*., 2012). They are known to play roles in fertilization by controlling the timing of pollen tube rupture (Miyazaki *et al*., 2009). Some members regulate the development of cell wall, such as *THESEUS1* (*THE1*) controlling lignin accumulation (Hematy *et al*., 2007).

**Figure 6:**
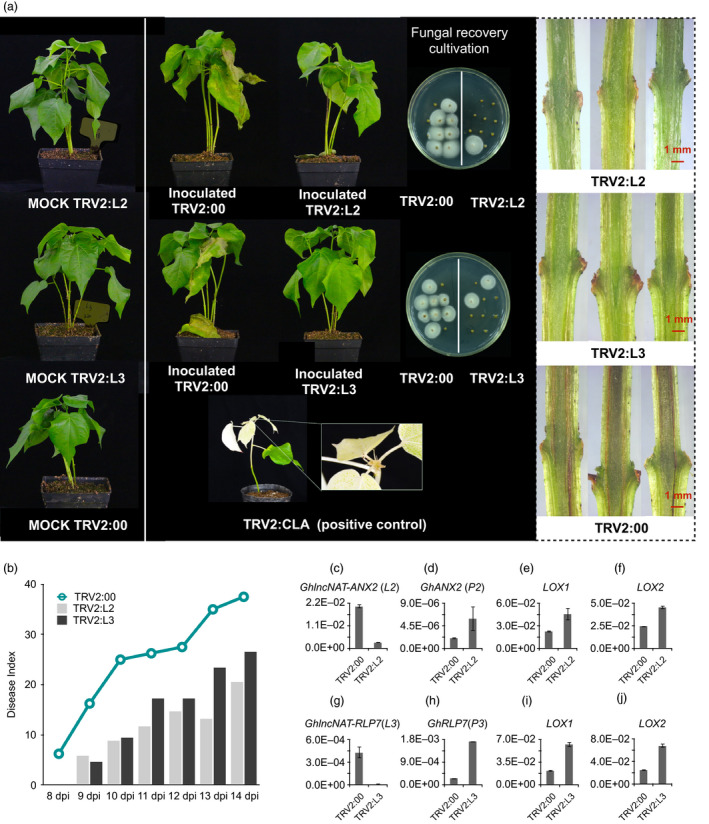
Functional identification of lncRNAs towards *Verticillium dahliae* in *Gossypium barbadense* using a virus‐induced gene silencing (VIGS) method. (a) Phenotypes of seedlings with lncNAT silencing postinoculation, showing the wilting phenotype, etiolated leaves, fungal recovery assay and stem inspection. *L2, GhlncNAT‐ANX2; L3, GhlncNAT‐RLP7*. *Cloroplastos alterados 1* (*CLA*) used as the positive control. (b) Disease index of infected plants. (c, g) The qRT‐PCR verification of *L2* (c) and *L3* (g) silenced by VIGS. (d, h) Expression change level of *P2* (d) after silencing *L2* and P3 (h) after silencing *L3*. (e, i) Transcriptional change of *lipoxygenase 1* (*LOX1*) after silencing *L2* (e) and *L3* (i). (f, j) Transcriptional change of *lipoxygenase 2* (*LOX2*) after silencing *L2* (f) and *L3* (j). Error bars show SDs (*n* = 3).

In this study, we explored the function of one core lncRNAs *GhlncNAT‐ANX2* (*L2*), involved in the plant–pathogen interaction, which was differentially regulated by pathogen. *L2* was firstly differentially up‐regulated at 6 hpi, and then, *P2* was later down‐regulated in 12 and 24 hpi in *G. hirsutum* after *V. dahliae* invasion (Figure S9). However, *L2* was slightly down‐regulated at 6 and 24 hpi in *G. barbadense* (Figure S9). We found that *L2*‐related protein‐coding gene and *GhANX2* (*P2*) belonged to the CrRLK1L family of RLKs, with the highest similarity with *ANX2* (Figure S10a). Virus‐induced gene silencing (VIGS) of *L2* plants showed enhanced resistance to *V. dahliae*, with reduced wilting and leaf defoliation (Figures 6a and S11a). Moreover, a fungal recovery assay on inoculated stem tissue showed reduced infection, and a reduced vascular browning phenotype also suggests an effect on infectivity (Figure 6a). The disease index (DI) and infected proportion of *L2*‐suppressed seedlings were sharply reduced compared to controls at all stages of *V. dahliae* infection (Figures 6b and S11b).

Cell surface‐located receptor‐like proteins (RLPs) have dual functions in plant development and immunity. Until now, only one locus (Ve1) has been shown to confer full resistance to *V. dahliae* and is also known as RLP (Fradin *et al*., 2009). Moreover, some homologs of *Ve1* from cotton may also be involved in disease resistance (Zhang *et al*., 2011, 2012). In this study, another core lncRNAs *GhlncNAT‐RLP7* (*L3*) involved in plant–pathogen interaction were differentially regulated when infected. *L3* was sharply up‐regulated at 6 and 12 hpi in *G. hirsutum*, but only slightly up‐regulated at 12 hpi in *G. barbadense* (Figure S9). In this study, *L3* paired protein‐coding gene *GhRLP7* (*P3*) was identified as *GhRLP7*, with the highest identity to *AtRLP7* in *Arabidopsis* (Figure S10b). *L3*‐silenced plants showed an enhanced resistance compared with the control, with less wilting and etiolated leaves (Figures 6a and S11a). In addition, fewer colonies in a fungal recovery assay and less browning of the vascular bundles were detected (Figure 6a). The DI and infection ratio also suggested enhanced resistance (Figures 6b and S11b).

After validation by efficient silencing of these target lncNATs, the expression changes of their paired neighbour protein‐coding genes were also checked (Figure 6c, d). Compared with the control, *P2* had a higher expression level in *L2*‐silenced plants (Figure 6d). Similarly, in *L3*‐silenced seedlings, *P3* was dramatically up‐regulated (Figure 6g,h). These results suggest that the influence of lncNATs on its neighbour protein‐coding genes seems to be negative.

Additionally, we detected the expression change of lipoxygenase 1 (*LOX1)* and lipoxygenase 2 (*LOX2)* both in *L2‐* and *L3*‐silenced seedlings. JA is a positive regulator of cotton immunity that regulates plant resistance to pests and pathogens (Gao *et al*., 2013, 2016; Rodriguez‐Saona *et al*., 2001), so the up‐regulation of JA pathway genes, like *LOX1* and *LOX2*, might contribute to the enhanced resistance in *L2*‐ and *L3*‐silenced plants (Figure 6e,f,i,j).

**Figure 7:**
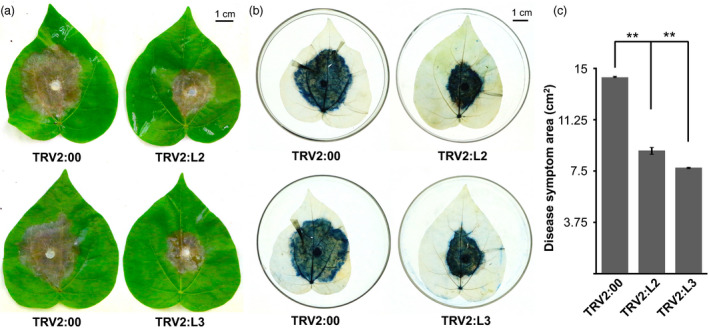
Functional identification of two lncRNAs towards *Botrytis cinerea* in *Gossypium barbadense* for virus‐induced gene silencing (VIGS) plants. (a) Disease symptoms of 3 days postinoculation leaves. (b) Trypan blue staining of hyphae cover area. (c) The statistics of disease symptom area. Error bars mean the standard deviation of three biological replicates. Asterisk represents statistically significant differences conducted by ANOVA test (**, *P *<* *0.01).

To further confirm our results, we performed the *in vitro* inoculation of *Botrytis cinerea* on cotton leaves. Consistent with *V. dahliae* inoculation results, both *L2‐* and *L3‐*silenced plants showed less necrosis, meaning an enhanced resistance towards *B. cinerea in vitro* (Figure 7a). The trypan blue staining results also supported this observation (Figure 7b). These findings were further supported by the statistics of symptom area, which showed a significantly smaller range of necrosis in both *L2‐* and *L3‐*silenced leaves (ANOVA, **P < 0.01) (Figure 7c).

## Discussion

The availability of allotetraploid cotton genome sequences provides extensive information for genic regions and their functional annotations. Noncoding regions, which comprise a large proportion of the genomes, have not previously been well characterized. A study on the expression of lncRNAs that have a 3′‐end poly(A) during cotton fibre development has been carried out (Wang *et al*., 2015b; Zhang *et al*., 2014b), but little is known about lncRNAs lacking a poly(A) tail. LncRNAs identified in the current study provide a comprehensive picture of non‐coding genomic regions in cotton.

Accumulating evidence suggests a link between lncRNAs and human disease (Wapinski and Chang, 2011). Nevertheless, there is limited information about plant disease‐related lncRNAs. In our research, the global expression contour of disease‐induced lncRNAs in allotetraploid cotton was firstly established. Intriguingly, the ratio of induced lncRNAs from the Dt subgenome was higher than that in the At subgenome in both cultivars ‘7124’ (*Verticillium*‐resistant *G. barbadense*) and ‘YZ‐1’ (susceptible *G. hirsutum*). Besides, more Dt‐biased homoeologous lincRNA pairs and Dt‐located VW resistance loci based on reference collection were found. This phenomenon suggests that a distinct disease response and function might exist in the two subgenomes, providing a possibility for functional divergence of homoeologous genes following polyploidization. Allotetraploid cotton is formed from two divergent A and D genomes (Wendel and Cronn, 2003; Wendel *et al*., 2012). Allopolyploidization leads to exclusive either At‐biased or Dt‐biased expression of homoeologous genes and asymmetric evolution of two subgenomes derived from different selective pressures (Yuan *et al*., 2015; Zhang *et al*., 2015b). Expression of A‐homoeologous positive selection genes (PSGs) is enriched during fibre elongation, while D‐homoeologous PSG expression is enriched in response to superoxide and other stresses (Zhang *et al*., 2015b). This predominant expression of the Dt subgenome in disease response provides a basis for understanding the evolution of the immune system in cotton.

Our research studied the distinct characters of species‐specific lncRNAs and species‐conserved lncRNAs in both cultivars. The comparison results unveiled genetic variation sources, which were mainly contributed by SNP but not TE insertion. The observed pathogen‐inducible expression and GO enrichment of LS lncRNAs suggest an important role of LS genomic regions in responding to disease. The GO analysis of core lncRNAs indicates their potential correlation with RNA methylation. RNA m(6)A methylation directs the translational regulation of abiotic stress response (Xiang *et al*., 2017; Zhou *et al*., 2015), but its roles in immunity have not been discovered and deserve future investigation.

Validating the functions of lncRNAs represents a major challenge in understanding RNA‐mediated gene regulation (Luo *et al*., 2016). Although thousands of lncRNAs have been identified from transcriptome profilings, the functions of the vast majority of them remain unknown (Luo *et al*., 2016). A variety of methods for investigating genomic location, chromatin features, tissue‐specific expression, subcellular localization and co‐expression of lncRNAs have been developed to predict and categorize their functions (Cabili *et al*., 2011; Luo *et al*., 2016; Mondal *et al*., 2010; Ponjavic *et al*., 2009). lncRNAs may function in modulating the transcription of their nearby genes (Luo *et al*., 2016). In this study, lncRNAs have been categorized into several functional groups based on annotation of nearby protein‐coding genes, which potentially helped predict unrecognized roles in response to pathogen inoculation. Therefore, we test expressional change of nearby protein‐coding genes and detected the significant up‐regulation when lncRNAs transcripts were silenced by VIGS, although more evidence should be provided. For example, chromatin isolation by RNA purification (ChIRP) and RNA immunoprecipitation (RIP) is necessary for elucidating this potential regulatory mechanism (Chu *et al*., 2011; Quinn *et al*., 2014).

This study represents the first to characterize the expression landscape of lncRNAs involved in plant responses to infection by *Verticillium* wilt. The enhanced resistance to *V. dahliae* and *B. cinerea* after silencing lncRNAs provides a possible road to improve the broad spectrum resistance towards multiple fungal pathogens. The identification of lncRNAs expressed in the context of plant defence may in the longer term provide new approaches for the genetic improvement of disease resistance traits in cotton. Future studies will be directed to understand the mechanism by which lncRNAs may regulate gene expression.

## Experimental procedures

### Plant material and fungal pathogen inoculation

Cotton seedlings of *G*. *barbadense* cv. 7124 and *G. hirsutum* cv. YZ1 were grown in vermiculite‐filled pots and watered with Hoagland's solution under greenhouse conditions of 25 °C for 2 weeks, under a photoperiod of 14‐h light and 10‐h dark. *V. dahliae* were cultivated in Potato Dextrose Agar (PDA) medium for 3–4 days from storage at −80 °C, and then, high activity hyphae were collected and then cultivated in Czapek's medium for 3 days at 25 °C. 10^6^ spores per mL in deionized water were used as the final concentration for inoculation.

When two fully expanded leaves appeared, whole plants were taken from the vermiculite for inoculation using a dipping infection method with the spores of *V. dahliae*, and the inoculated plants were returned to the pots. Roots were harvested at 6, 12 and 24 h postinoculation. Plants treated with distilled water were collected at different time points for use as mock treatments. All samples were stored at −80 °C until further use.

### Stranded RNA library construction and sequencing

High‐quality RNA was extracted using a guanidine thiocyanate method (Zhu *et al*., 2005). The stranded libraries only removing rRNAs were constructed using the Ribo‐Zero Kit (Illumina, San Diego, CA) following the manufacturer's instructions. Sequencing was performed on the Illumina Hiseq™ 2000 system in the Beijing Genomic Institute. The regular stranded libraries in which only mRNAs with poly(A) tails retained were constructed using Illumina TruSeq Stranded RNA Kit (Illumina) and performed on the Illumina Hiseq™ 2000 system.

### LncRNA identification and classification

All sequence data were firstly processed by filtering the low‐quality reads (the ratio of base with *Q* < 10 should be >50% of whole read) and adapters. Reads derived from rRNA were removed by SOAP alignment. We mapped those reads to the cotton genome (*G. hirsutum* L. cv. TM‐1) by applying TOPHAT (Trapnell *et al*., 2009; Zhang *et al*., 2015b). Each transcriptome was assembled separately by CUFFLINKS, while background noise was filtered based on Fragments Per Kilobase of transcript per Million base pairs sequenced (FPKM), length, coverage and status threshold (FPKM > 0.5; length > 200; coverage > 1; status: OK) (Trapnell *et al*., 2010). The separated gene models from the same cultivar were merged together using the CUFFMERGE procedure. Novel transcripts were detected by CUFFCOMPARE. The coding potential capability was calculated by Coding Potential Calculator (value < 0). Finally, the class code ‘u’ represents the long intergenic noncoding RNAs (lincRNAs), class code ‘x’ represents long noncoding natural antisense transcripts (lncNAT), class code ‘j’ represents the sense transcripts, and class code ‘i’ represents the intronic transcripts. The lincRNA/protein‐coding gene pairs were restricted to nearby 5 kb regions and nonoverlapping with 1 kb away from protein‐coding genes.

### Identification of species‐common (core) and species‐specific (LS) lncRNAs

As mentioned above, all separated transcriptome gtf files of *G. barbadense* were merged into one gtf file using CUFFMERGE with parameter ‐g. Simultaneously, all individual transcriptome gtf files of *G. hirsutum* were merged following the same procedure. These merged transcriptomes from *G. barbadense* and *G. hirsutum* made it possible to compare the loci of lncRNAs from different cultivars using CUFFCOMPARE. The class code ‘u’ represents the specific lncRNAs between *G. barbadense* and *G. hirsutum*. Beyond this, sequences with similarity were discarded to ensure the reliability of identified specific lncRNAs according to reciprocal BLASTN results with E threshold (*E* value < 1e‐10). The class code ‘=’ represents the core lncRNAs between *G. barbadense* and *G. hirsutum* that share fully equal loci. A reciprocal BLASTN (*E* value < 1e‐10) was also run to improve the confidence of identified core lncRNAs, and only those with high sequence similarity were retained for further analysis.

### Expression analysis

We applied CUFFMERGE to merge multiple assemblies to get merged transcripts separately for two cotton cultivars. The expression of all identified lncRNAs was processed by CUFFDIFF, and genes expressed differentially were obtained by the following criteria: adjusted *P* value < 0.001 and at least twofold FPKM change (Trapnell *et al*., 2010). The expression of lncRNAs was normalized and then clustered into several groups by *K*‐means in Gene Expression Similarity Investigation Suite software (Genesis; http://genome.tugraz.at/genesisclient/genesisclient_description.shtml).

### GO enrichment analysis

All GO terms of listed genes were annotated using Blast2GO (https://www.blast2go.com) by comparing to the reference genome background (*P *<* *0.01).

### Phylogenetic analysis

Protein sequences were aligned by Clustalx (http://www.clustal.org). Phylogenetic trees were constructed using an unweighted paired‐group method with arithmetic means (UPGMA) followed by a bootstrap test in MEGA4 (http://www.megasoftware.net/mega4/) and visualized in FigTree (http://tree.bio.ed.ac.uk/software/figtree/).

### Virus‐induced gene silencing (VIGS) vector construction and genetic transformation

The lncNATs and their paired protein‐coding genes always have overlapping regions with each other, so it is essential to investigate the genomic organization and the overlap between them. We designed specific primers to amplify fragments (avoiding overlapping and conserved regions) to construct VIGS vectors as indicated in the scheme design to ensure the silencing specificity for each gene (Figure S12). Nonoverlapping regions were identified by genic genomic locations; nonconserved regions were found according to the NCBI Conserved Domain Search web service (http://www.ncbi.nlm.nih.gov/Structure/cdd/wrpsb.cgi). Primer pairs that were used to construct vectors are provided in Table S3. The preparation of TRV vectors and *Agrobacterium tumefaciens* in experiments was conducted as previously reported (Fradin *et al*., 2009; Gao *et al*., 2013). The fragment of candidate genes armed with infusion connections was separately inserted into the TRV:00 vectors. Positively ligated plasmids were transformed into *A. tumefaciens* GV3101. TRV1 vectors were, respectively, mixed with TRV vectors that comprised candidate genes or empty vector TRV:00 using equal amounts, and then, agro‐infiltration by syringe was used to infiltrate 10‐day‐old seedling cotyledons of *G*. *barbadense* cv. 7124 (Gao *et al*., 2013). TRV:CLA1 (*CLOROPLASTOS ALTERADOS 1*) was utilized as the positive control and the empty vector TRV:00 as the negative control.

### VIGS plant inoculation and fungal recovery assay

Two weeks after infiltration, the bleaching phenotype of positive controls appeared. Then, we started to prepare inoculation. The seedlings were pulled out of the pot carefully, and then, the plant roots were dipped into the distilled water with 10^5^ spores per litre for 1 min. After that, each four inoculated seedlings were re‐planted in one larger pot. We performed the inoculation with at least 16 plants for each treatment using *V. dahliae* isolate V991 with at least three biological replicates. Disease index (DI) for plant populations was calculated as previously described (Gao *et al*., 2013). The higher score the population had, the lower the resistance. Similarly, the rate of diseased plant was used to estimate the susceptibility of the whole population.

After inoculation by *V. dahliae* for 2 weeks, the fresh stems on cotyledon nodes were collected from the same position on each plant and sterilized by 84 disinfectants for 5 min. After washing 3 times by sterilized water, disinfected edges were removed and the stems were cut into small pieces. Stem samples were inoculated on PDA medium and cultured at 25 °C for 3–5 days. The fungi in stem were inspected by light microscopy (Leica MZFLIII, Wetzlar, Germany).

Similarly, we conducted the inoculation of *B. cinerea* when bleaching phenotype of positive control appeared. *B. cinerea* for leaves inoculation was cultivated at 25 °C for 3–5 days. Only outermost part was utilized to guarantee the high pathogenicity of fungi. Then, leaves with *B. cinerea* were cultivated in 25 °C for 3 days; then, the disease symptom area in each leaf was calculated in photographs. Staining of leaves by trypan blue was boiled in lactophenol‐trypan blue for 15 min for the first step and then destained by chloral hydrate overnight as previously described (Gao *et al*., 2013).

### Real‐time (RT) PCR analysis

Total plant RNA was extracted from cotton root using a guanidine thiocyanate method. The first stranded cDNA was synthesized from 2 μg RNA using the M‐MLV reverse transcript system (Promega, Fitchburg, Wisconsin). We designed gene‐specific primers (design strategy was similar as mentioned above) to conduct the qRT‐PCR verifications as indicated in the scheme design to ensure measure specificity for each gene (Figure S12). Quantitative real‐time (RT) PCR was run at 95 °C for 3 min followed by 28–35 cycles at 95 °C for 20 s, 55–60 °C for 20 s and 72 °C for 20 s. Quantitative RT‐PCR was conducted on an ABI 7500 Real Time PCR system (Applied Biosystems, Waltham, Massachusetts) with the iTag™ Universal SYBR^®^ Green Supermix (Bio‐Rad, Hercules, California). Gene expression levels were normalized to *UB7* expression (Tan *et al*., 2013).

### Data access

The stranded RNA‐seq data generated from *G. barbadense* and *G. hirsutum* were submitted to NCBI Sequence Read Archive database with the BioProject ID PRJNA360482.

## Conflict of interest

The authors have declared that no competing interests exist.

## Supporting information


**Figure S1** Summarized data for sequenced samples.
**Figure S2** Summary of reported genetic mapping results about *Verticillium* wilt resistance loci.
**Figure S3** Distribution of pearson correlation coefficient for putative paired and random pairs.
**Figure S4** The global expression profiles of lncRNAs in *G. hirsutum*.
**Figure S5** Distribution of transposon elements overlapping with or located within lincRNAs and lncNATs.
**Figure S6** SNP distribution of lineage‐specific (LS) lncRNAs and core lncRNAs.
**Figure S7** Functional implication of differentially induced pairs of lincRNAs and lncNATs.
**Figure S8** Examples of plant pathogen interaction pathways that candidate genes are involved in.
**Figure S9** Expression validation and correlation between qRT‐PCR and transcriptomic analysis.
**Figure S10** Phylogenetic trees of candidate lncNAT‐paired protein coding genes.
**Figure S11** Phenotypes and proportion statistics of infected plants.
**Figure S12** The genomic location and scheme design of primers for verifying lncRNAs and protein‐coding genes.

**Table S1** Distribution of identified lncRNA number in two types of stranded libraries.

**Table S2** *Verticilium* wilt resistance loci in cotton modified from references.

**Table S3** List of PCR primers used in this study.
