# Supplementary material for: Long noncoding RNAs involve in resistance to Verticillium dahliae, a fungal disease in cotton
Source: Plant Biotechnol J. 2017 Dec 21;16(6):1172–85. doi: 10.1111/pbi.12861 (PMC5978870; doi:10.1111/pbi.12861)
Supplement: Supplementary file 1 — Figure S1 Summarized data for sequenced samples. Figure S2 Summary of reported genetic mapping results about Verticillium wilt resistance loci. Figure S3 Distribution of pearson correlation coefficient for putative paired and random pairs. Figure S4 The global expression profiles of lncRNAs in G. hirsutum. Figure S5 Distribution of transposon elements overlapping with or located within lincRNAs and lncNATs. Figure S6 SNP distribution of lineage‐specific (LS) lncRNAs and core lncRNAs. Figure S7 Functional implication of differentially induced pairs of lincRNAs and lncNATs. Figure S8 Examples of plant pathogen interaction pathways that candidate genes are involved in. Figure S9 Expression validation and correlation between qRT‐PCR and transcriptomic analysis. Figure S10 Phylogenetic trees of candidate lncNAT‐paired protein coding genes. Figure S11 Phenotypes and proportion statistics of infected plants. Figure S12 The genomic location and scheme design of primers for verifying lncRNAs and protein‐coding genes. [file PBI-16-1172-s003.pdf]

Sample Name	Clean reads	Genome map Rate	Gene map Rate	Expressed Gene	Expressed Transcripts	Expressed Exon	Novel Transcripts	Extend Gene	Alternative Splicing	SNP	Indel
H12m	124091008	79.12%	49.69%	27469	27469	246576	15591	18482	98481	281440	5575
H12v	120172446	79.27%	50.26%	33326	33326	250798	15712	20029	110660	266338	6009
H24m	119694042	77.91%	48.85%	34160	34160	255209	16094	20865	114861	281481	6014
H24v	120358500	78.16%	49.75%	32264	32264	250510	15981	19038	111043	273762	5580
H6m	120545390	80.93%	50.12%	29542	29542	251989	15337	19480	106882	263763	5569
H6v	120232226	80.81%	47.34%	27254	27254	251282	14805	19394	103769	249353	4732
Y12m	124105058	81.57%	51.28%	29511	29511	253911	15871	20662	95788	108545	944
Y12v	120092764	78.32%	49.18%	32439	32439	255694	18454	20932	105495	127329	1315
Y24m	124010650	80.08%	50.88%	29327	29327	247502	15417	19177	92026	123975	990
Y24v	123836684	79.70%	49.18%	28930	28930	244923	14969	19374	90365	117831	960
Y6m	120152676	81.23%	52.86%	32107	32107	257301	15497	21466	98518	97656	937
Y6v	120588960	82.24%	52.08%	32473	32473	258127	19448	21118	112817	148694	1386

Figure S1 Summarized data for sequenced samples. Summary of stranded RNA sequencing and mapping using the *Gossypium hirsutum* genome as reference (Zhang *et al.*, 2015b). “H” represents the species *G. barbadense* and “Y” represents *G. hirsutum*. The numbers “6”, “12”, “24” indicate the number of hours post-treatment. “m” and “v” indicates roots that were treated separately with water or *Verticilium dahliae*.

The distribution of Verticillium wilt resistance loci in cotton

Chr	Number	Chr	Number
A01	4	D01	4
A02	2	D02	3
A03	6	D03	7
A04	7	D04	4
A05	13	D05	14
A06	3	D06	2
A07	7	D07	17
A08	12	D08	6
A09	8	D09	18
A10	3	D10	5
A11	7	D11	9
A12	2	D12	8
A13	3	D13	0
Total (A)	76	Total (D)	97

Figure S2 Summary of reported genetic mapping results about *Verticillium* wilt (VW) resistance loci. "Chr" indicated cotton chromosomes. "Total(A)" indicated the total numbers of VW resistance loci located in At subgenome. "Total(D)" indicated the total numbers of VW resistance loci located in Dt subgenome. These results indicated the biased distribution in subgenomes of VW resistance loci.

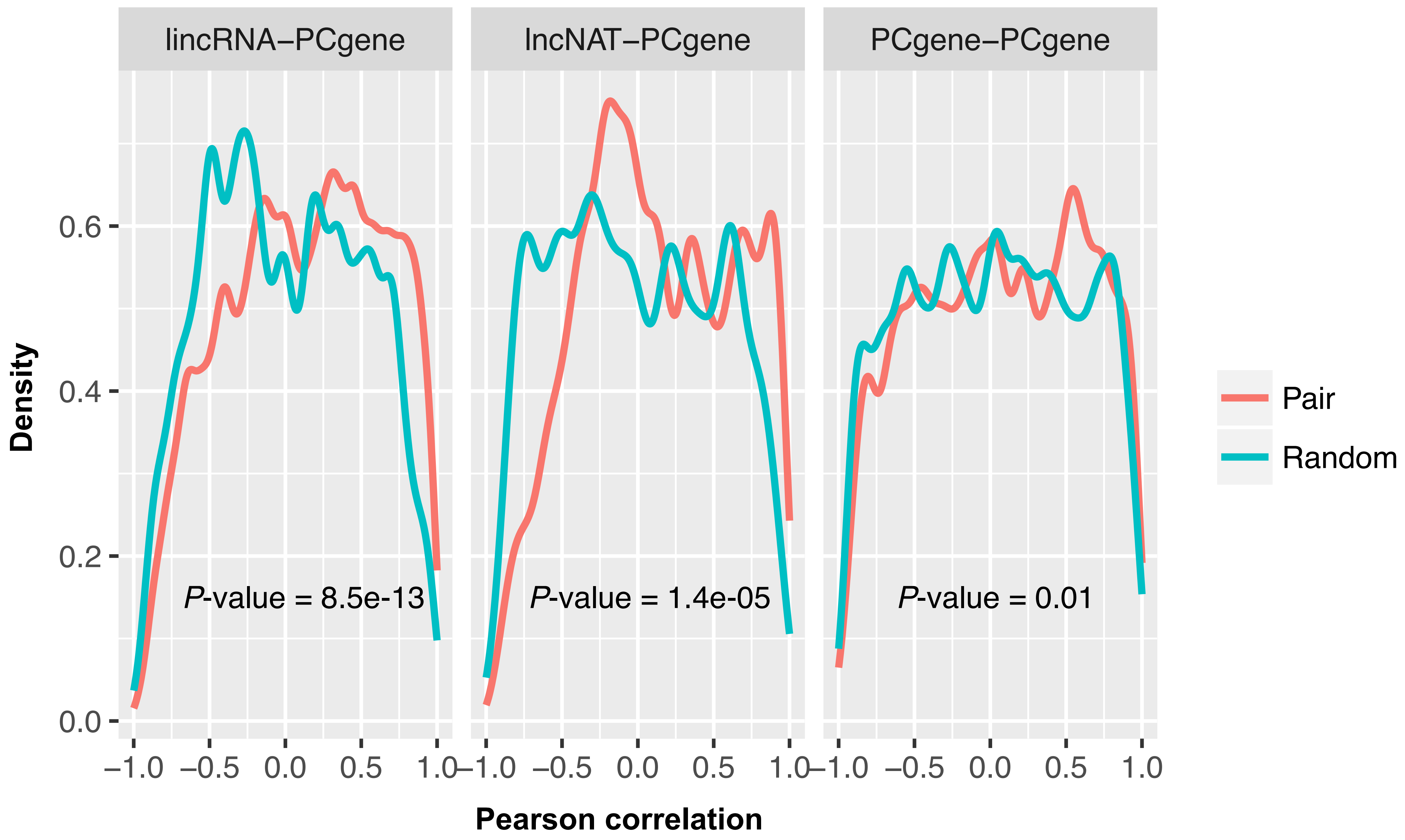

Figure S3 Distribution of pearson correlation coefficient for putative paired and random pairs.The red and green lines mean density of expression correlation between the pair and random gene pairs. Significant levels of distribution divergence are indicated as *P* values.

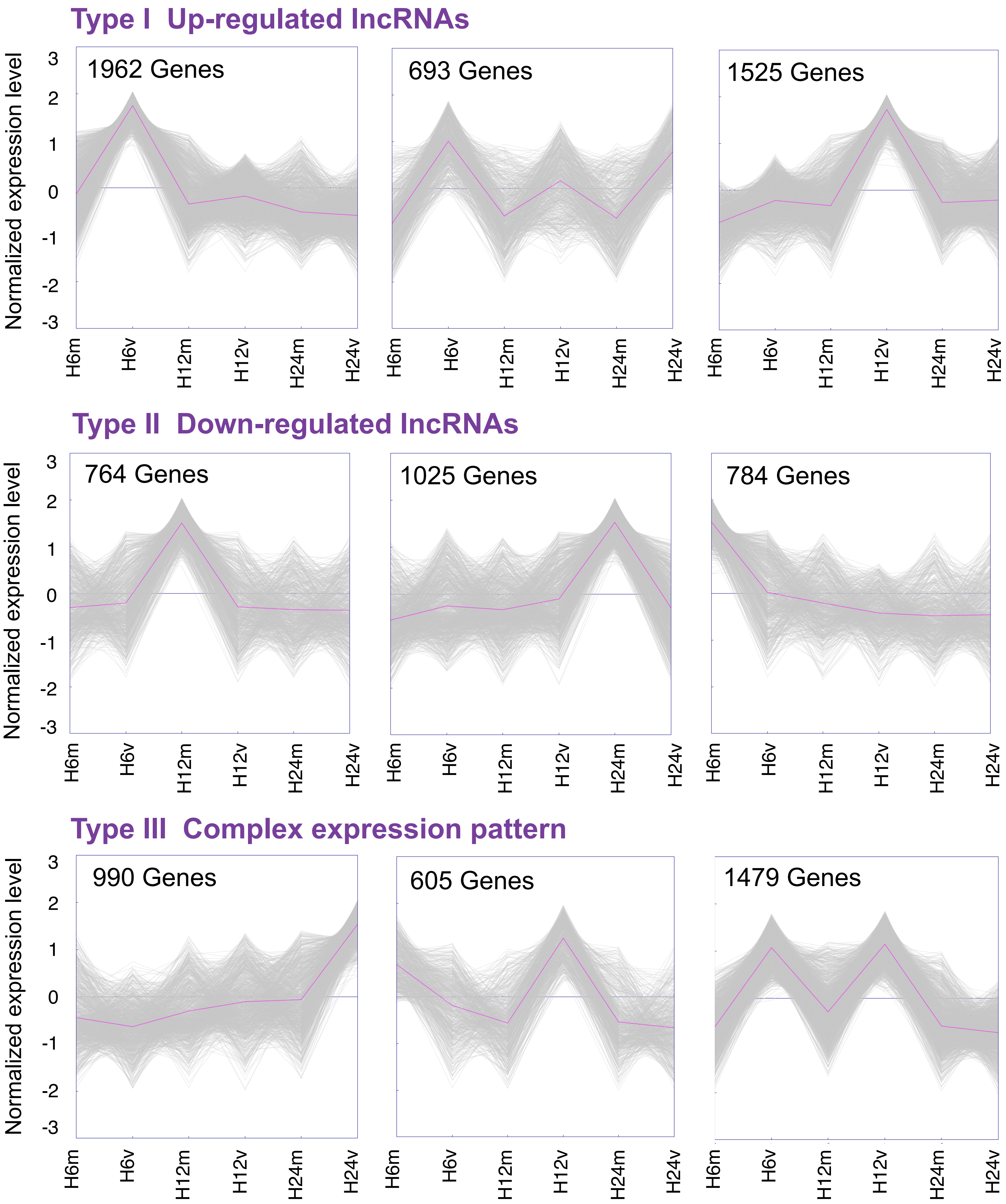

Figure S4. The global expression profiles of lncRNAs in *G. hirsutum*. Clusters of expressed lncRNAs were developed by K-means. “6”, “12” and “24” means hours post infection. “m” and “v” mean mock and seedling roots inoculated with *Verticillium dahlia*e V991.

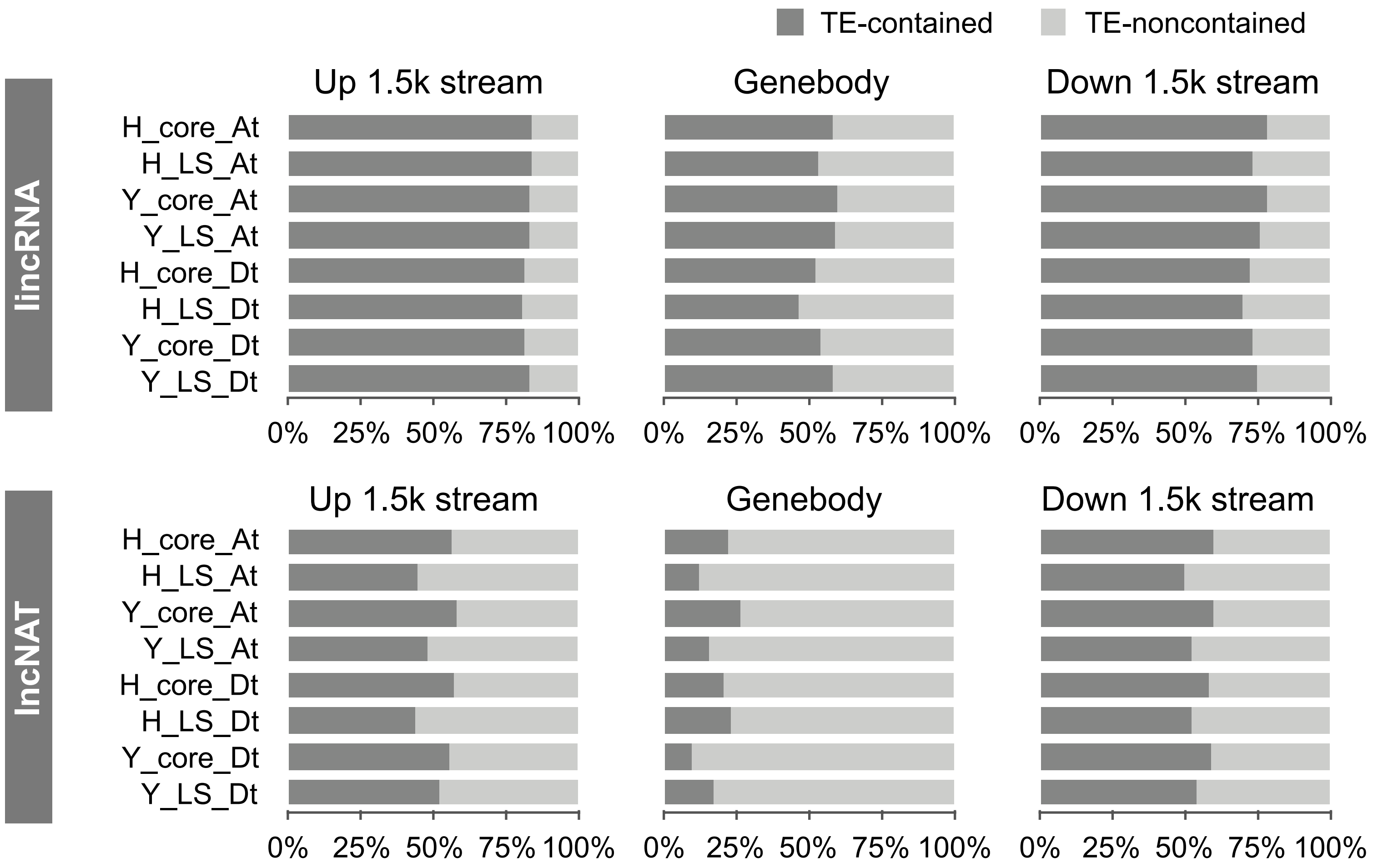

Figure S5 Distribution of transposon elements overlapping with or located within lincRNAs and lncNATs. Upper and lower three plots show the statistics of TE content in lncRNAs in genic upstream 1.5K bp region, genic region, downstream 1.5 kb region. Dark grey shows lncRNAs containing TEs, while light grey shows lncRNAs containing no TEs. “H” and “Y” represent *G. barbadense* and *G. hirsutum.* “Core” and “LS” mean core and lineage specific (LS) lncRNAs. “At” and “Dt” mean the At subgenome and the Dt subgenome.

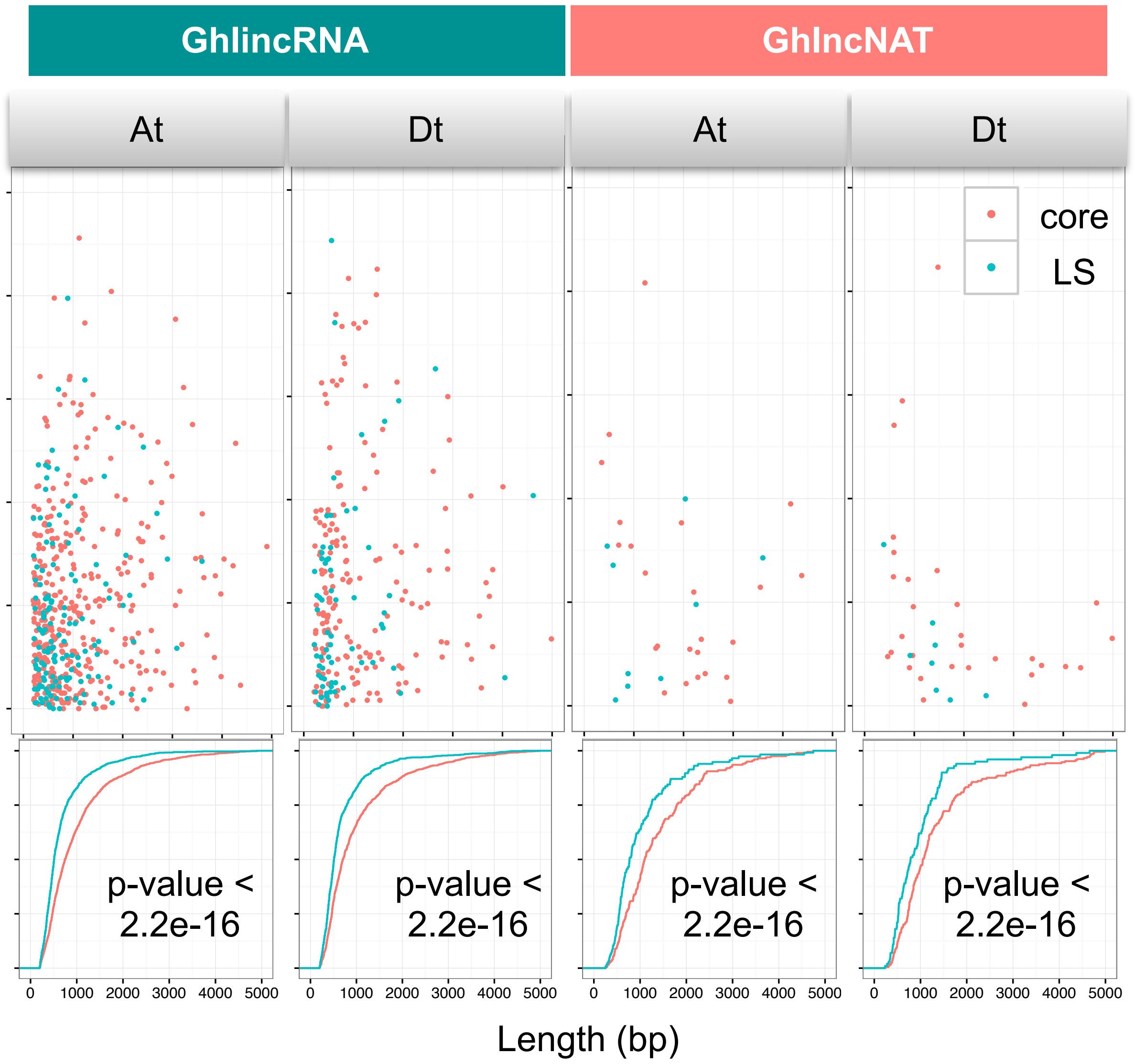

Figure S6 SNP distribution of lineage specific (LS) lncRNAs and core lncRNAs. Scatter plot showing the correlation between SNP frequency and length of lncRNAs in *G. hirsutum*. Red and green points represent core and LS lncRNAs. The lower plots show comparisons of accumulated SNP frequency distribution against length of core and LS lncRNAs in red and green. Significant levels of distribution divergence are indicated as *P* values.

(a)

(b)

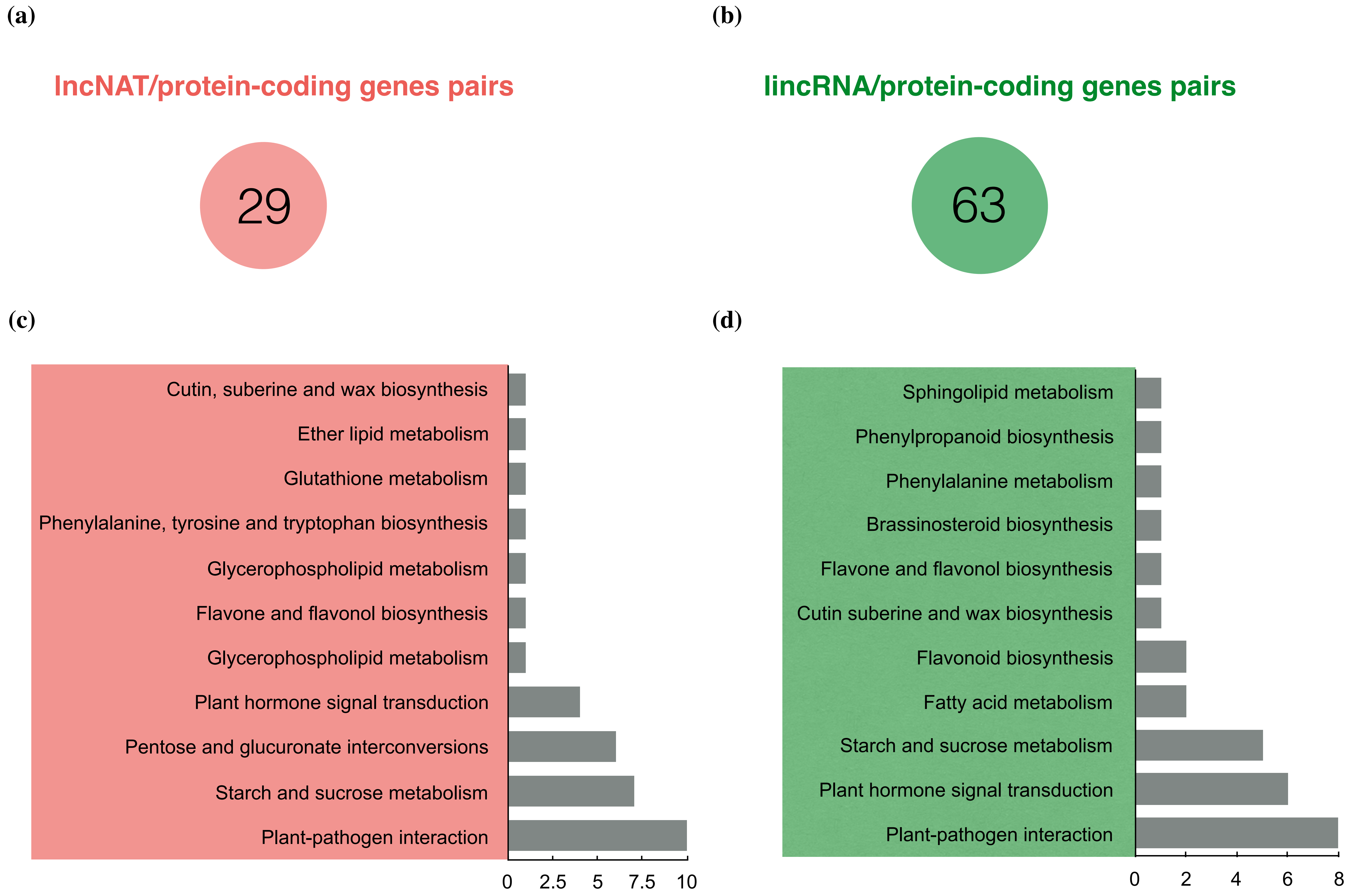

Figure S7 Functional implication of differentially induced pairs of lincRNAs and lncNATs. (a) The number of lncNAT/protein-coding genes pairs. (b) The number of lincRNA/protein-coding genes pairs. (c) gene ontology (GO) of lncNAT/protein-coding genes pairs. (d) gene ontology (GO) of lincRNA/protein-coding genes pairs. X axis represents the gene number. GO is shown to illustrate the pathways in which differentially regulated genes participate.

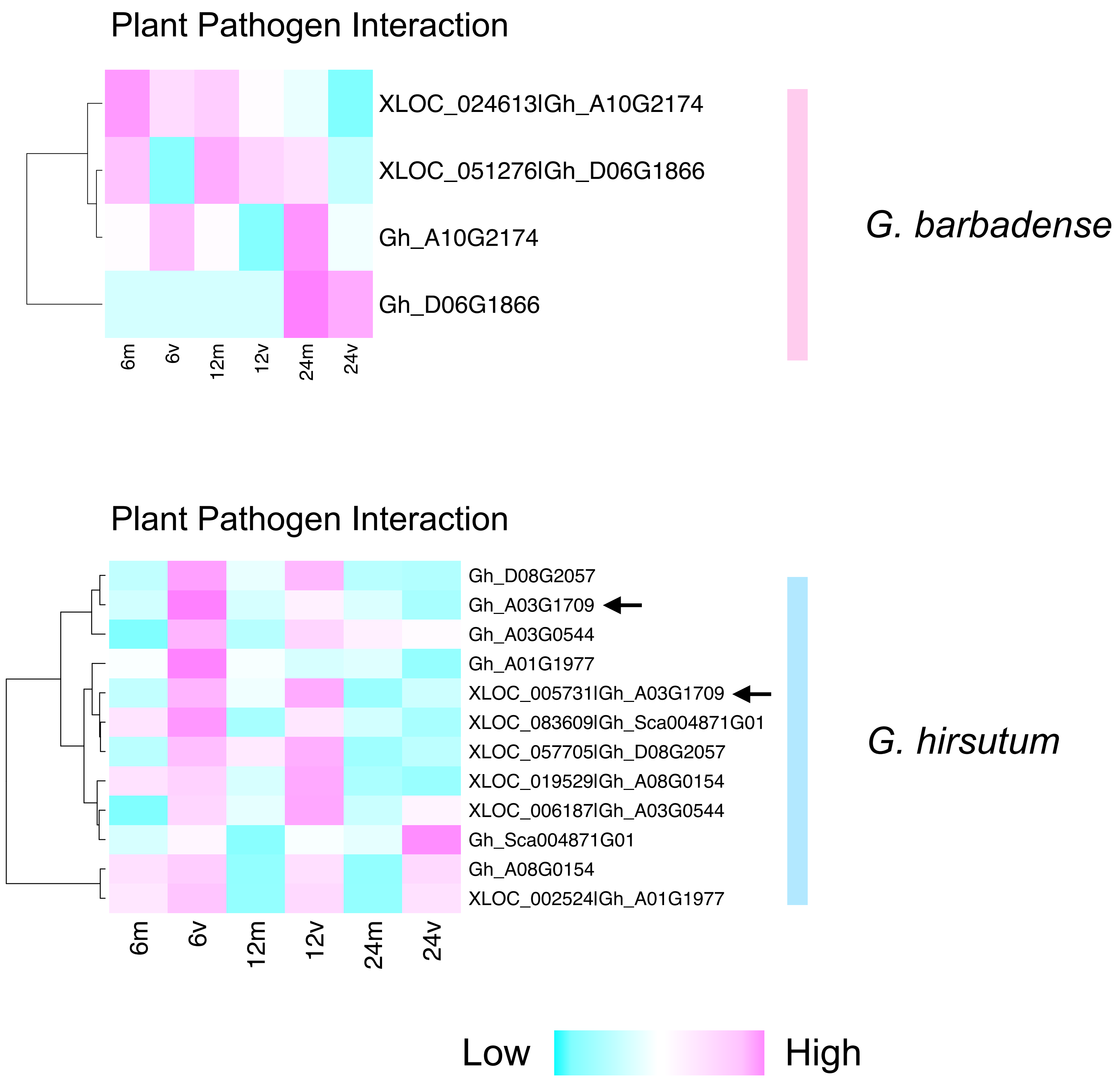

Figure S8 Examples of plant pathogen interaction pathways that candidate genes are involved in. The numbers “6”, “12”, “24” below the heatmaps indicate the hours post treatment. The “m” and “v” indicate which roots were treated either with water or *Verticilium dahliae*.

(a)

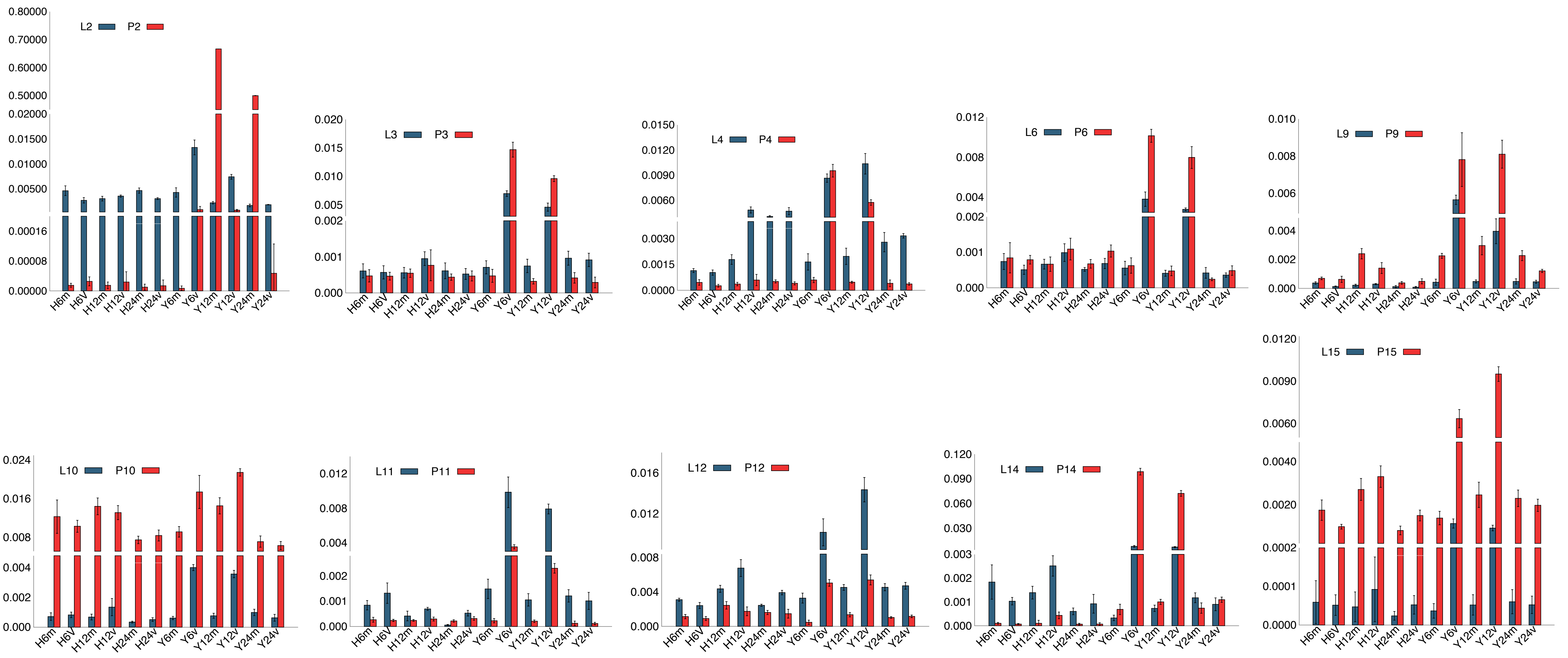

(b)

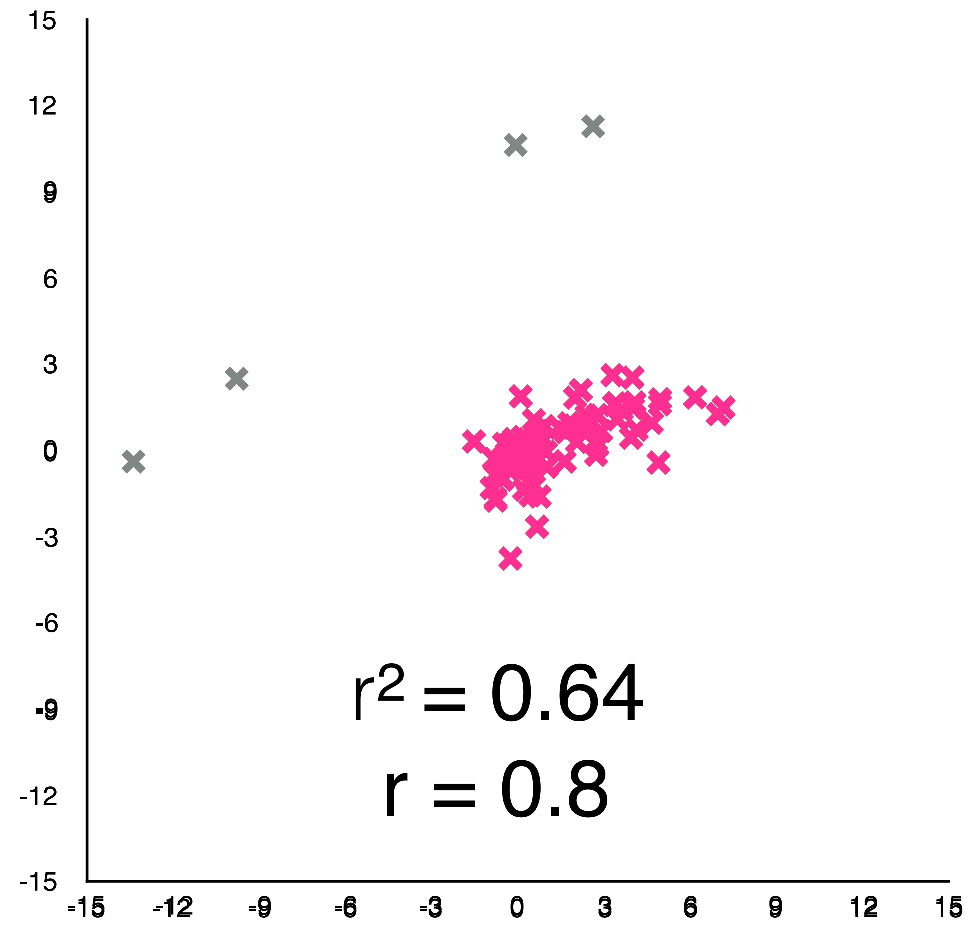

Figure S9 Expression validation and correlation between qRT-PCR and transcriptomic analysis. (a) Expression validation by qRT. The Y-axis represents the relative expression changes of candidate genes that are normalized with UB7 (Tan et al., 2013). For the X-axis, "H" represents the species G. barbadense and "Y" represents G. hirsutum. The numbers "6", "12", "24" indicated the hours post-treatment. "m" and "v" indicate which roots were treated either with water or Verticilium dahliae. (b) Correlation between qRT-PCR (X-axis) and FPKM from sequencing data (Y-axis) between the selected ten pairs of lncRNAs and protein-coding genes.

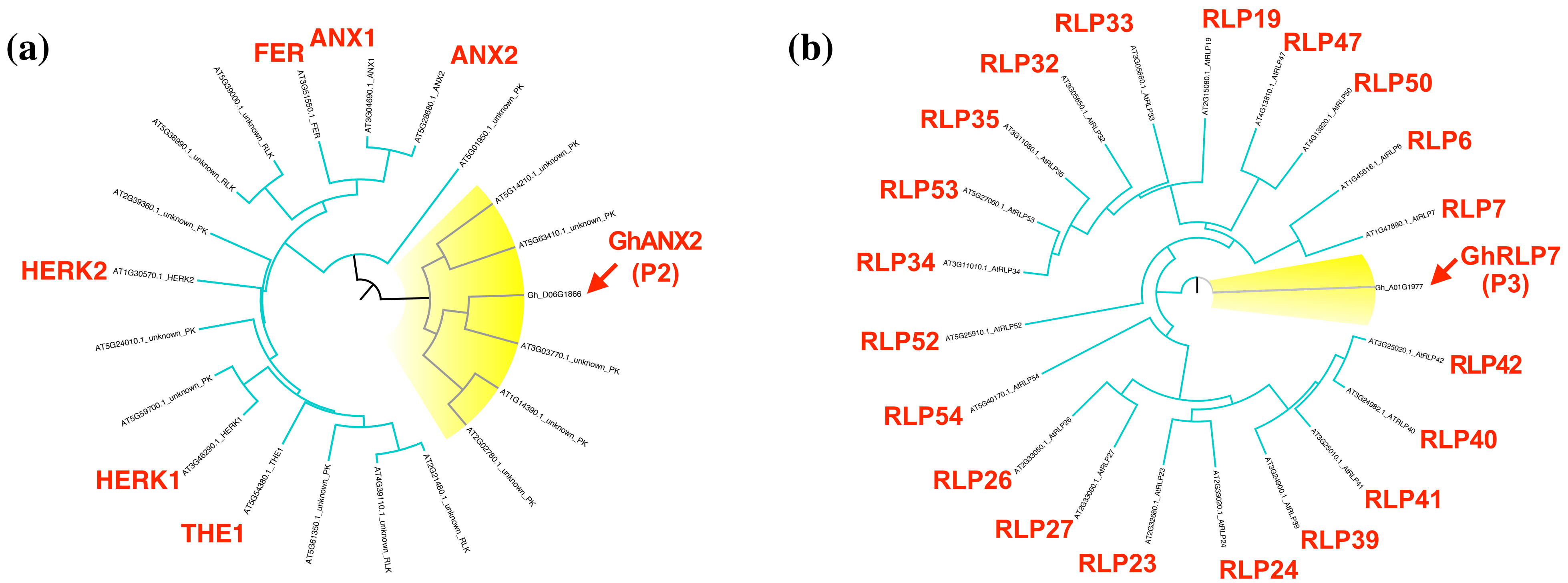

Figure S10 Phylogenetic trees of candidate lncNAT-paired protein coding genes. (a) The relationship between *P2* and previously reported genes according to BLAST result (E-value < 1e-5). *P2* is shown near *AtANX2*. (b) *P3* is closest to *AtRLP7*.

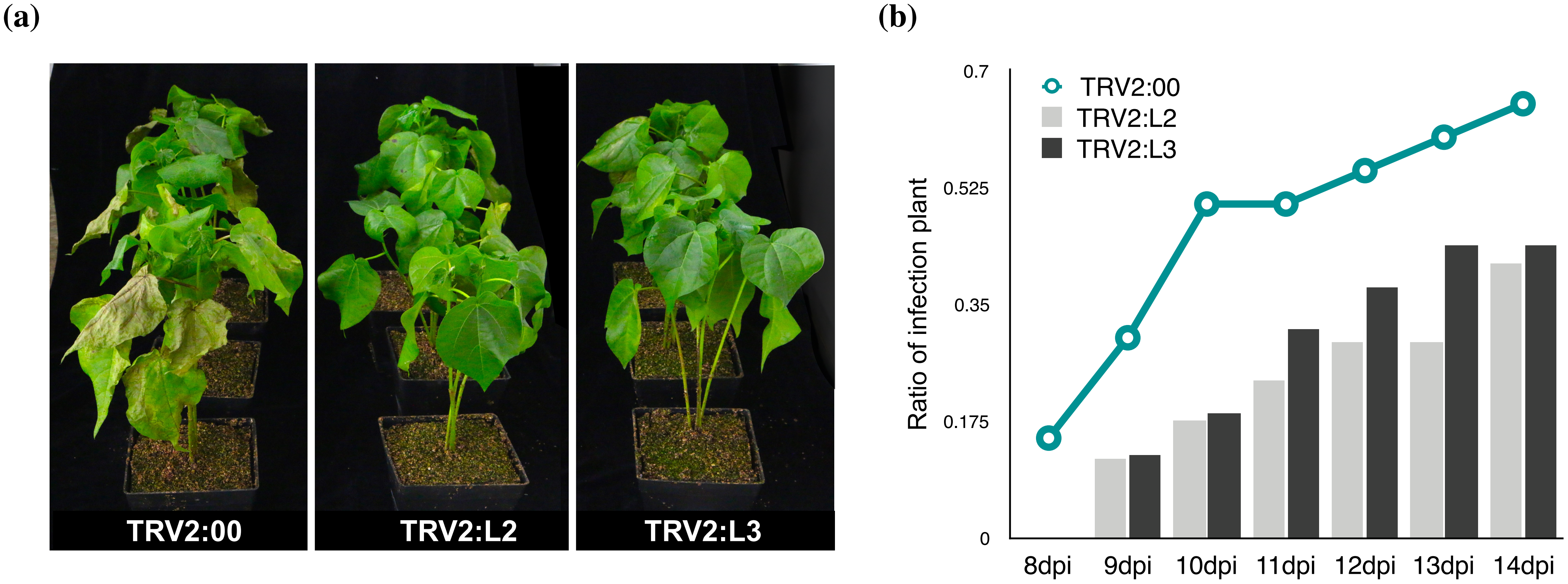

Figure S11 Phenotypes and proportion statistics of infected plants. (a) phenotypes of infected plants after VIGS. (b) Statistics analysis of seedlings infected ratio after inoculated with *Verticillium dahliae*. The Numbers from "8" to "14" represent days post infection (dpi).

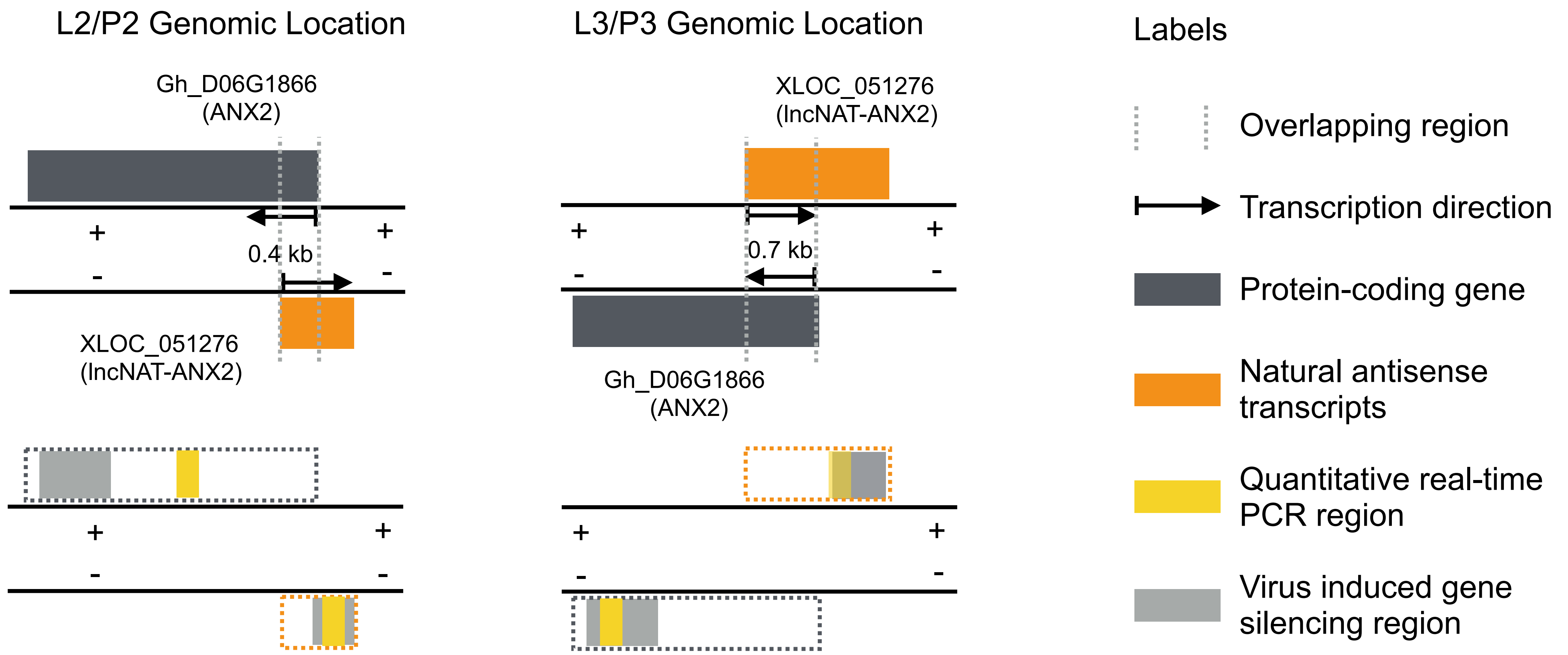

Figure S12 The genomic location and scheme design of primers for verifying lncRNAs and protein-coding genes. Specific primers (avoiding overlapping and conserved regions) were designed to ensure the silencing specificity and reliability for each target.
